# Effect of Cathode Microstructure on Electrochemical Properties of Sodium Nickel-Iron Chloride Batteries

**DOI:** 10.3390/ma14195605

**Published:** 2021-09-27

**Authors:** Byeong-Min Ahn, Cheol-Woo Ahn, Byung-Dong Hahn, Jong-Jin Choi, Yang-Do Kim, Sung-Ki Lim, Joon-Hwan Choi

**Affiliations:** 1 Korea Institute of Materials Science (KIMS), Changwon 51508, Gyeongnam, Korea; vhehtkfkd123@kims.re.kr (B.-M.A.); cera72@kims.re.kr (B.-D.H.); finaljin@kims.re.kr (J.-J.C.); 2 Department of Materials Science and Engineering, Pusan National University, Busan 46241, Korea; yangdo@pusan.ac.kr; 3 Department of Material Chemistry and Engineering, Konkuk University, Seoul 143-701, Korea; sklim@konkuk.ac.kr

**Keywords:** sodium nickel chloride, iron, battery, cell capacity, microstructure, low cost, durability, NaCl size, granule density, cycle retention

## Abstract

Sodium metal chloride batteries have become a substantial focus area in the research on prospective alternatives for battery energy storage systems (BESSs) since they are more stable than lithium ion batteries. This study demonstrates the effects of the cathode microstructure on the electrochemical properties of sodium metal chloride cells. The cathode powder is manufactured in the form of granules composed of a metal active material and NaCl, and the ionic conductivity is attained by filling the interiors of the granules with a second electrolyte (NaAlCl_4_). Thus, the microstructure of the cathode powder had to be optimized to ensure that the second electrolyte effectively penetrated the cathode granules. The microstructure was modified by selecting the NaCl size and density of the cathode granules, and the resulting Na/(Ni,Fe)Cl_2_ cell showed a high capacity of 224 mAh g^−1^ at the 100th cycle owing to microstructural improvements. These findings demonstrate that control of the cathode microstructure is essential when cathode powders are used to manufacture sodium metal chloride batteries.

## 1. Introduction

Battery energy storage systems (BESSs) are an indispensable technology due to the global growth in electric energy consumption. Although various types of BESSs are available [1,2,3,4], most BESSs consist of lithium ion batteries (LIBs) because of their many advantages, such as high energy density and efficiency. However, in the BESS field, size enlargement of LIBs has proven difficult, and they also exhibit a safety issue. Na/NiCl_2_ batteries are promising alternatives to other types of BESSs as they overcome these problems; that is, they are safer and easier to scale up than LIBs. Furthermore, Na/NiCl_2_ batteries offer merits such as a high energy density and low cost. However, commercial Na/NiCl_2_ cells have poor price competitiveness because of the large amount of Ni they contain and their high operation temperature. Because of these weaknesses, many experimental strategies for improving Na/NiCl_2_ batteries have been attempted, such as replacing the Ni in the cathode with other metals [5,6,7,8,9,10], decreasing the operating temperature [11,12,13,14] and enhancing Ni-based cathodes [15,16,17,18]. However, the cathode microstructure, which is essential for in-depth cathode studies on topics such as the NaCl size effect or granule density, has not been researched in detail. Nonetheless, other studies have examined the influence of increased NaCl size after cycling under various conditions [12]. However, this result was related to the effects of cell degradation. Additionally, although the effect of NaCl size on Na/AlCl_3_ batteries was briefly studied [6], its effect on Na/NiCl_2_ and Na/(Ni,Fe)Cl_2_ batteries remains unidentified. Moreover, the effects of the granule manufacturing process have been studied [19], but this study mainly analyzed the granule production conditions, whereas the microstructure was not examined in detail.

**Figure 1:**
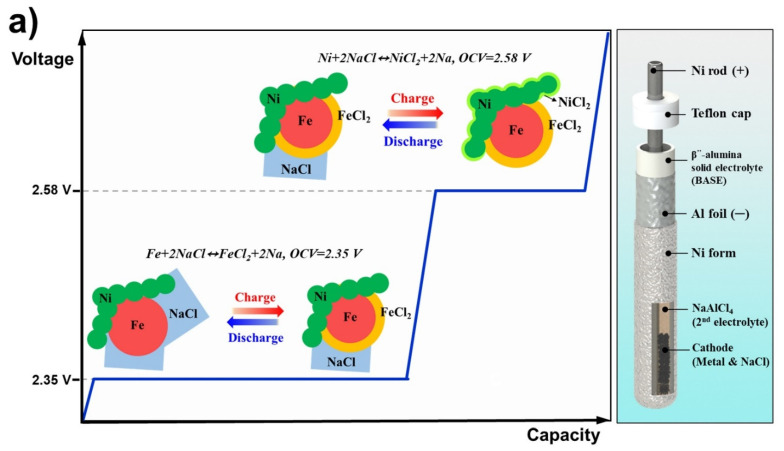

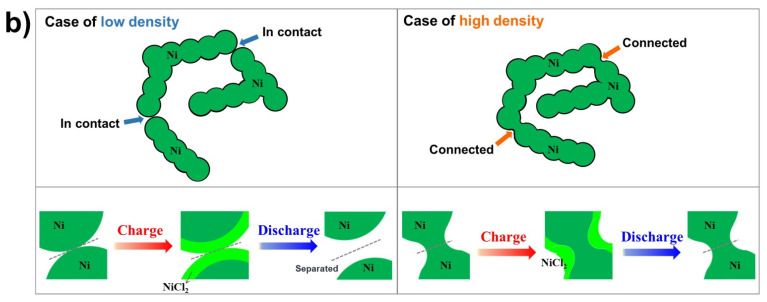
Schematic diagrams. (**a**) charge–discharge mechanism of Na/(Ni,Fe)Cl_2_ cell and simple test cell structure; (**b**) difference in the Ni connection shape between low and high densities.

We previously studied microstructure control in Na/(Ni,Fe)Cl_2_ batteries [17]. A method of control of the Ni and Fe particle sizes was used to improve the electrochemical properties of the cell with an increase in the connectivity between the Ni particles. Further, a Na/(Ni,Fe)Cl_2_ battery with an optimized Ni/Fe ratio was fabricated [18], which demonstrated the properly divided roles of the Fe and Ni particle. Specifically, the Fe particles mainly contributed to the charge–discharge process, and the Ni particles maintained electron paths with minimized electrochemical reaction, as shown in the schematics of Figure 1a. In addition, decreasing the Ni content (Ni/Fe = 1/1, metal/NaCl = 1.5/1) lowered the cost, and the cells thus constructed were more durable than Na/NiCl_2_ batteries. However, previous Na/(Ni,Fe)Cl_2_ cells showed a problematic decline in capacity; owing to a lack of additional microstructure optimization except for Ni and Fe size control, the cell capacity after 50 cycles decreased below 200 mAh g^−1^ (per weight of Ni–Fe particles). Thus, in this study, we first considered the density of the cathode granules, which is a fundamental aspect of the microstructure, before examining the effect of the NaCl size in the cathode materials. We divided the NaCl size into three ranges (<38, 38–150 and 150–300 μm) and the granule density into two levels (low density: 10 MPa, high density: 200 MPa). Additionally, we investigated the correlation between the microstructure and electrochemical properties by controlling the NaCl size and granule density to prevent the capacity of the Na/NiCl_2_ cells from declining. Consequently, the NaCl size and granule density were optimized to improve the electrochemical properties of the modified Na/(Ni,Fe)Cl_2_ battery.

## 2. Experimental Section

### 2.1. Preparation of Cathode Granules

NaCl (98%, Sigma Aldrich, Burlington, MA, USA) was sieved and classified into three size grades: small (<38 μm), medium (38–150), and large (150–300 μm). For the cathode of the Ni cell, three grades of NaCl powders were each separately mixed with raw Ni powder (99.7%, 2.5 μm, Standard Grade, Vale) by ball milling without balls for 2 h. The Ni–NaCl mixtures were prepared with a Ni/NaCl ratio of 2/1. A 1:1 mixture of Ni and Fe (99.5%, 6.5 μm, BASF, Ludwigshafen, Germany) was mixed with a Turbula-type T2C shaker mixer (Willy A. Bachofen AG, Uster, Switzerland) for 2 h to reinforce the connection between the metal powders. The mixing process for the metal and NaCl in the Ni–Fe cell was the same as that of the Ni cell. The cathode of the Ni–Fe cell used a metal/NaCl ratio of 1.5/1. The granule density was measured using Archimedes’ method. Although this method is inaccurate because of the pores penetrating the granule surface, it can be used to simply compare the densities of the two processes. For comparison, the densities in this study were thus expressed as relative densities (%). The granule density was divided into two conditions, low and high. Low-density granules were pressed at 10 MPa and had a relative density within the range of 28–37%. High density granules were pressed at 200 MPa and had a density of 52–56%. The pressed sheet was ground using a granulator, and the granule size was 300 μm to 1 mm.

### 2.2. Synthesis Method and Additives of Second Electrolyte

The NaAlCl_4_ (the 2nd electrolyte) was synthesized using NaCl and AlCl_3_ (99.985%, Alfa Aesar, Ward Hill, MA, USA) powders at 300 °C for 1 h. For ideal crystallization, the synthesized liquid NaAlCl_4_ was cooled in a furnace. After the cooling step, solid NaAlCl_4_ was ground using a mortar with approximately 5 wt.% sulfur powders (reagent grade, −100-mesh, Sigma Aldrich, Burlington, MA, USA) to remove the oxide layers on the metal surface in the cathode [19].

### 2.3. Configuration of a Simple Test Cell

Figure 1 shows the schematic of a simple test cell. The β^”^-alumina solid electrolyte (BASE; Ionotec, Berkeley, UK) was wrapped with Al foil and fixed using the Ni form. The Al foil played two roles: a place for storing the Na melt and a current collector on the anode side. The cathode current collector was made of a Ni rod, and the upper side of the cell was sealed with a Teflon cap to prevent contamination. The anode side of BASE tube was coated with carbon paste (Research Institute of Industrial Science and Technology (RIST), Pohang-si, Korea) to improve the wettability of Na melt to the BASE surface [20]. The BASE tube was heat-treated at 230 °C for 30 min after carbon coating to remove a polymer and water contained in the carbon paste.

### 2.4. Test Condition of Cells

All test cells were set up at 300 °C in the electric furnace which was located inside the Ar-filled glove box. Moisture and oxygen concentration were maintained at less than 1.0 ppm. The Ni-only cells were cycled using VMP3 (Bio Logic SAS, Seyssinet–pariset, France) between 2.4 V and 2.8 V, and the C-rate was set to C/2. The 1.5LNF-low cell was cycled between 2.2 V and 2.7 V and the C-rate was set to C/8. The microstructure of cathode was observed using a scanning electron microscope (SEM, IT-300; JEOL CO., Tokyo, Japan). Slightly different conditions were applied to the 1.5SNF-high cell compared to the 1.5LNF-low cell, except the operation temperature and cut-off voltage (2.2–2.7 V). The C-rate was controlled with five steps. Initially, the first step was cycled at 34 mA g^−1^ to the 4th cycle for stabilization. The second and third steps increased the current density double and triple, respectively, for 5 cycles at each step due to the examination the rate capability. Additionally, the fourth step was set up at a slow rate of 34 mA g^−1^ again to the 19th cycle. Lastly, for the verification of cycle retention, the cells were cycled at 67 mA g^−1^ to the 100th cycle. The characterization of the microstructure is the same as for other cells.

## 3. Results and Discussion

### 3.1. Electrochemical Effects of Granule Density and NaCl Size in the Na/NiCl_2_ Cells

We divided the granule into high-density and low-density according to the pressure, and performed an electrochemical test with three sizes of NaCl for each density. The microstructure of the low-density granules is shown schematically in Figure 1b, wherein agglomerates of Ni particles are simply in contact. During the initial cycle, this contact provided sufficient interparticle connectivity. However, as charge–discharge proceeded, a NiCl_2_ layer was repeatedly generated and destroyed on the Ni surface; eventually, Ni particles that were previously only in contact became separated. Since these separated Ni particles could not participate in the electrochemical reaction, the capacity decreased as the cycle proceeded. Conversely, in high-density granules formed under a higher pressure, the Ni agglomerates were well connected between particles. This strong connectivity was maintained even after charge–discharge cycling due to the NiCl_2_ layer that was created only on the Ni surface. This Ni connectivity was a key factor in the cycle retention of the high-density cells.

**Figure 2:**
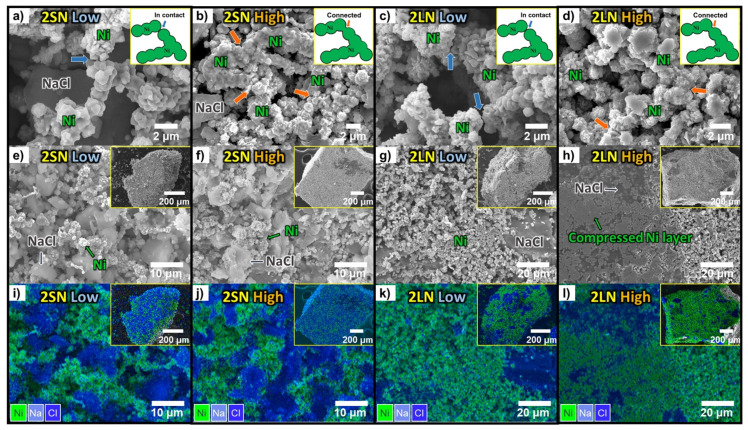
SEM images of cathode granule in 2SN and 2LN with different granule densities. (**a**–**d**) shape of Ni connection in each condition; (**e**–**h**) granule surface and microstructure of each cell; (**i**–**l**) EDS mapping data of each cell. (green: Ni, blue: NaCl).

The difference in granule density was confirmed using the SEM images in Figure 2. Figure 2a,c shows that in the low-density granules, Ni particles were simply in contact (blue arrows). By contrast, the strong connections frequently existed in the high-density granule microstructure (red arrows). In fact, the microstructure of each cell was similar to the corresponding schematic diagram in Figure 1b. However, the microstructure of the high-density granules also showed a disadvantage when observed at a lower magnification. For excellent battery operation, the cathode must have sufficient porosity for the liquid electrolyte to fully penetrate [21]. For 2SN-low in Figure 2e,i and 2SN-high in Figure 2f,j, both of which used small NaCl, the microstructure of the high-density granule seemed slightly dense, which means that the 2SN-low granules that have a wider contact area with the second electrolyte were favorable for a high initial capacity. The 2LN-low and 2LN-high cells showed similar tendencies. However, when the NaCl surface was large, the Ni layer was compressed on this surface owing to the high pressure. This compressed Ni layer may be another factor that interrupts the contact between the second electrolyte and NaCl, in addition to granule porosity.

**Figure 3:**
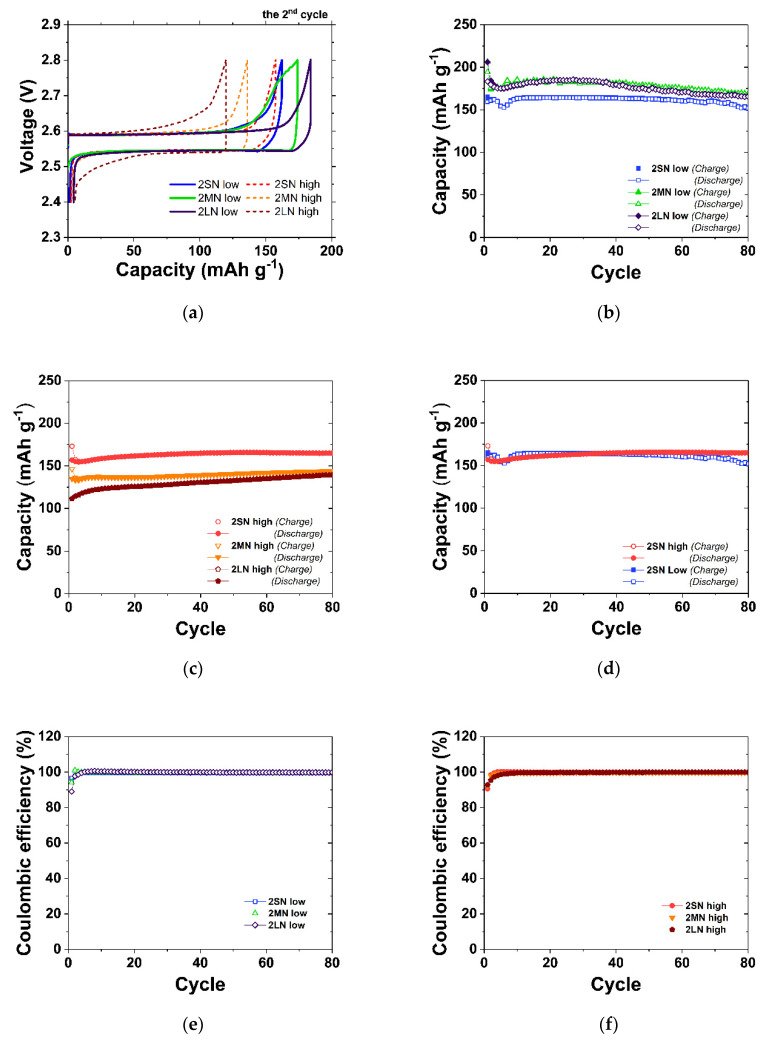
Electrochemical behavior of Na/NiCl_2_ batteries. (**a**) Voltage profile of each cell at the second cycle; (**b**) cycling performance of low-density Ni cells to the 80th cycle; (**c**) cycling performance of high-density Ni cells to the 80th cycle; (**d**) comparison of cycling performance between 2SN-high and 2SN-low cells; (**e**,**f**) Coulombic efficiency.

Figure 3a shows the voltage profiles of all Ni cells at the second cycle, and as expected, the low-density cells indicated higher initial capacities than the high-density cells. The initial capacity of the low-density cells ranged from 165 to 184 mAh g^−1^. Thus, compared with the high-density cells (120–157 mAh g^−1^), the low-density granules were more favorable to a high initial capacity. As mentioned above, the high porosity of low-density granules facilitates the penetration of the second electrolyte into the granules; hence, the activation volume of low-density granules is higher than that of high-density granules. The initial capacity differences of 2LN, 2MN and 2SN cells, which have different densities, were 64, 38 and 8 mAh g^−1^, respectively. Particularly in the case of LN cells, which had the widest capacity difference, contact with the second electrolyte was inhibited not only by the factor of porosity, but also the compressed Ni layer. Because of these two features, the initial capacity of 2LN-high showed the lowest value of 120 mAh g^−1^. When small NaCl was used for the Na/NiCl_2_ cell, the difference in the initial capacity was the smallest. The 2SN-high granules did not exhibit this compressed Ni layer, and among all the Na/NiCl_2_ cells, the cells made with small NaCl particles showed the most similar microstructure and initial capacity according to the difference in density.

The density clearly affected the cycling performance of the cells, as shown in Figure 3b,c. Although the low-density cells had a higher initial capacity than the high-density cells, the capacity began to decrease after 26 cycles for 2MN-low and 2LN-low and after 40 cycles for 2SN-low. The 2SN-low showed the lowest initial capacity of 165 mAh g^−1^ among the low-density cells, resulting in a thinner layer of NiCl_2_ on the Ni surface. The thinner NiCl_2_ layer tends to maintain the Ni connection more easily after discharge [18]. Therefore, although it started low, the initial capacity of 2SN-low was maintained for a relatively longer period than that of the other low-density cells. Notably, among the low-density cells, the capacity of 2MN-low cell at the 80th cycle unexpectedly showed the highest value. However, because the two cells had almost the same maximum capacity (2MN-low: 186 mAh g^−1^/2LN-low: 185 mAh g^−1^) or 80th cycle capacity (as shown in Table 1), this difference was not very noticeable. Conversely, high-density cells showed the opposite tendency. First, the capacity of high-density cells was properly maintained to the 80th cycle. As shown in Figure 1b, the strong Ni connectivity provided high cycle retention. In 2MN-high and 2LN-high, the capacity increased as the charge–discharge cycling proceeded; it seemed that the activation volume was gradually expanded. However, since the increased capacity at the 80th cycle was a low-level capacity of 143–144 mAh g^−1^, the capacities of 2MN-high and 2LN-high cannot be an advantage. The most notable high-density cell was the 2SN-high cell. As shown in Figure 3c, the initial capacity of this cell was 8 mAh g^−1^, lower than that of 2SN-low; however, as cycling proceeded, its capacity increased to 165 mAh g^−1^ at the 80th cycle. At the 80th cycle, the capacity of 2SN-high was similar to those of 2MN-low and 2LN-low, and if the capacities of 2MN-low and 2LN-low had decreased with the same tendency after the 80th cycle, the capacity of 2SN-high would be higher than that of the two other corresponding cells. The coulombic efficiencies of all Ni cells were almost 100% except for the initial charge–discharge section. In summary, the high initial capacity and weak connectivity between Ni particles in the low-density cell were unfavorable to the cycling life. However, among high-density cells with strong Ni connectivity, 2MN-high and 2LN-high cells were not suitable due to their low capacity. Finally, because the 2SN-high cell had a similar capacity to the low-density cells and exhibited a high cycle retention, small NaCl and high-density granules were selected as the optimal conditions for application in the Na/(Ni,Fe)Cl_2_ cell.

### 3.2. Application of Small NaCl on the Na/NiCl_2_ and Na/(Ni,Fe)Cl_2_ Cell of Metal/NaCl = 1.5/1

**Figure 4:**
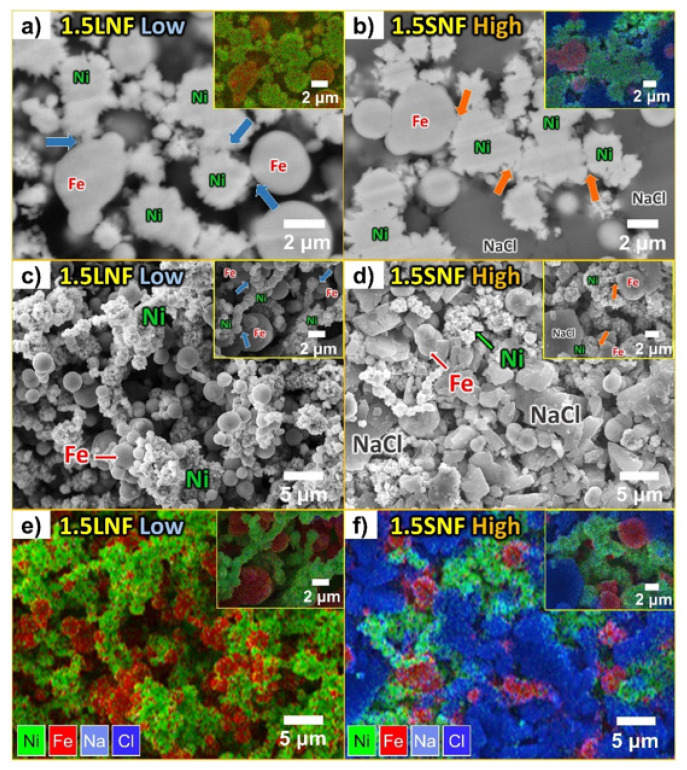
SEM images of cathode granules. (**a**) backscattering image for contacting Ni and Fe particles in 1.5LNF-low granules; (**b**) backscattering image for well-connected Ni and Fe particles in 1.5SNF-high granule; (**c**,**e**) microstructure of the 1.5LNF-low cell and Ni–Fe connections similar to those of the 2LN-low cell; (**d**,**f**) microstructure of the 1.5SNF-high cell and strong connections among Ni and Fe particles.

In our previous Ni–Fe composite cells, such as 1.5LNF-low, the cathode granules had been made by milling the cathode powder without pressure. Therefore, the density of 1.5LNF-low granules was lower than that of low-density Ni cells in this study. For comparison with 1.5LNF-low cells, small NaCl and high-density granules, which had been the optimal conditions in the Ni-only cell test, were applied to the new Ni–Fe composite cell. The backscattering image of Figure 4a,b shows the connection difference of the two Ni–Fe composite cells. Through the shape of particle cross-sections, the difference in connectivity between the two cells was confirmed more clearly. As seen in Figure 4c,d, both Ni–Fe cells contained well-connected Ni–Fe composites. The difference in the microstructure according to the density is similar to that of the Ni-only cells. Many contact points appeared between the active metal particles in the 1.5LNF-low granules (inset of Figure 4c). Conversely, in the 1.5SNF-high microstructure shown in Figure 4d, the active metal particles were strongly connected, as in the high-density Ni cells. In addition, the microstructure of 1.5SNF-high was much denser than that of 1.5LNF-low due to the high pressure.

**Figure 5:**
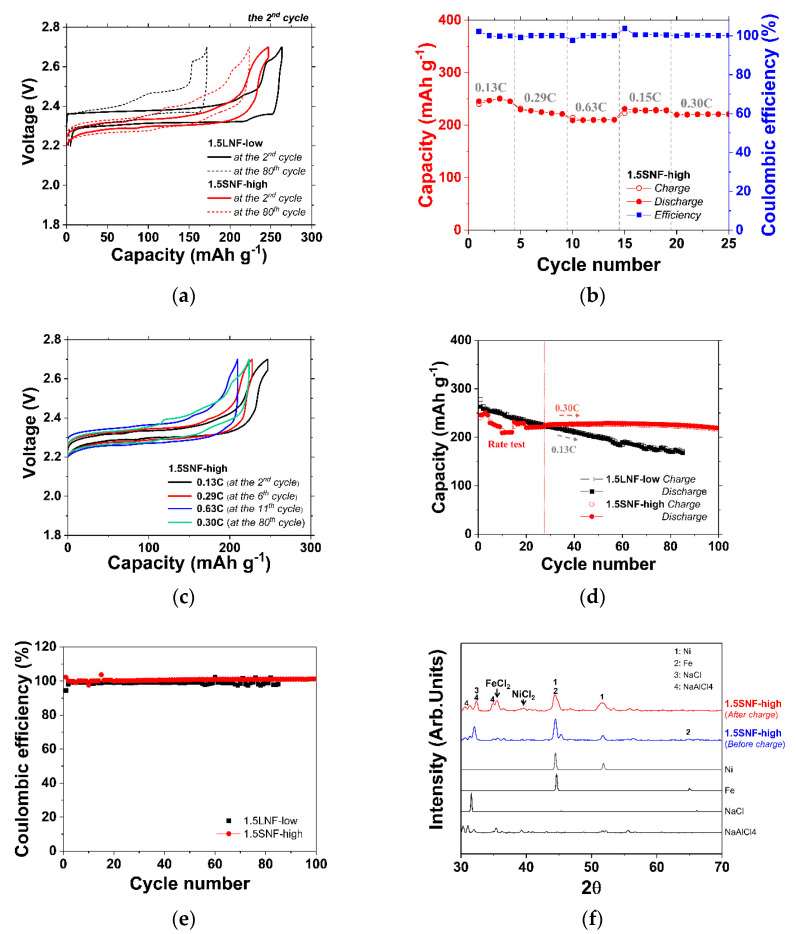
Electrochemical properties of 1.5LNF-low and 1.5SNF-high. (**a**) voltage profile; (**b**) rate capability of 1.5SNF-high cell; (**c**) voltage profile according to C-rate; (**d**) cycle performance for 100 cycles; (**e**) Coulombic efficiency; (**f**) X-ray diffraction (XRD) spectra.

As shown in the voltage profile of Figure 5a, in both Ni–Fe composite cells, the roles of Ni and Fe were properly divided according to the ratio of Ni/Fe = 1/1. Specifically, most of the capacity was acquired through the Fe reaction, and approximately 10% of the Ni reacted. This small amount of Ni reaction forms a thin NiCl_2_ layer, which has an advantage in that it conserves the Ni connection as a conduction path. The capacity of both cells was increased by changing the metal/NaCl ratio to 1.5/1. The theoretical capacities of 1.5SNF-high and 1.5LNF-low are 314 mAh g^−1^; that is, higher than the theoretical capacities of Ni-only cells (306 mAh g^−1^ on the ratio of metal/NaCl = 1.5/1) due to Fe particles being lighter than Ni particles. The initial capacities, which also followed the tendency of Ni-only cells, were 263 and 247 mAh g^−1^ for 1.5LNF-low and 1.5SNF-high, respectively. In both cells, the Fe reaction was decreased at the 80th cycle. In particular, the Fe reaction of 1.5LNF-low declined significantly. On the other hand, the Ni reaction was largely constant between the second cycle and the 80th cycle. Prior to the cycle retention test, the stepwise rate capability test of the 1.5SNF-low cell was conducted at five different current densities over 25 cycles in order to confirm the high-speed charge–discharge capability (as shown in Figure 5b). The starting current density was 34 mA g^−1^, which was doubled (67 mA g^−1^) and quadrupled (134 mA g^−1^) in the second and third steps, respectively. In the first to third steps, the charge–discharge capacity was higher than 200 mAh g^−1^ without a considerable capacity loss.

When the rate was returned to the initial current density in step four, the charge capacity did not fully return to the initial capacity. However, in the last step of 67 mA g^−1^, the charge capacity returned to the same capacity as that in the second step. Figure 5c shows the voltage profile for each step. As the C-rate increased, the charge–discharge voltage in the Fe plateau was increased. However, the portion of Ni reaction was constant at the various C-rates, and only the Fe reaction showed a tendency to decrease. In the SNF-high at the 80th cycle, the Fe reaction decreased and the Ni reaction was slightly increased; as a result, the capacity remained near the initial capacity.

The cycling properties in 1.5SNF-high were tested at a higher C-rate (of 0.30 C) than 1.5LNF-low (0.13 C) to rapidly determine its cycling life properties. Nevertheless, 1.5SNF-high showed outstanding performance and higher stability during high-speed charge–discharge. Compared with Ni-only cells with the same metal/NaCl ratio, 1.5LNF-low also showed significantly better cycle retention owing to the Ni–Fe composite microstructure [18]. However, the microstructure of the 1.5LNF-low cell had many simple contacts between metal particles—thus, the electronic conduction path was disconnected by the formation of a chloride layer, and the capacity consistently decreased from the initial capacity. As shown in Figure 5d, the capacity of 1.5LNF-low decreased from 263 mAh g^−1^ at the second cycle to 172 mAh g^−1^ at the 80th cycle, thus 1.5LNF-low retained 65% of initial capacity to the 80th cycle. On the other hand, 1.5SNF-high continued to stabilize until the 46th cycle after the rate test, and its capacity slightly increased relative to that at the end of the rate test. Similar to the results of the Ni cells, the internal spaces in 1.5SNF-high that allowed the second electrolyte to penetrate were narrower than those of 1.5LNF-low owing to the small NaCl and high pressure. Because of these narrow internal spaces, 1.5SNF-high had a lower initial capacity than 1.5LNF-low. However, as discussed earlier in terms of the microstructure, 1.5SNF-high showed high cycle retention due to the strong connection between metal particles. The initial capacity of 1.5SNF-high was 247 mAh g^−1^ at 0.13 C, and the capacity at the 80th cycle was 224 mAh g^−1^ at 0.30 C, although the current density increased to twice that of 1.5LNF-low. Furthermore, 1.5SNF-high maintained its capacity from the 80th cycle to the 100th cycle, thereby retaining 91% of its initial capacity after the 100th cycle. As seen in Figure 5e, the coulombic efficiency of 1.5SNF-high also remained stable at approximately 100%, except for the cycle in which the current density was changed during the rate test. Figure 5f shows the XRD spectra of raw powder and cathode materials in the 1.5SNF-high. After charging, the FeCl_2_ peak showed high intensity compared to the NiCl_2_ peak. Therefore, it can be explained that the cathode granule was manufactured as intended. In summary, in the case of the 1.5SNF-high cell, which contained an optimized microstructure, the initial capacity was 16 mAh g^−1^, lower than the previous condition of 1.5LNF-low. However, 1.5SNF-high showed a high capacity of 224 mAh g^−1^ to the 100th cycle and a high coulombic efficiency of 100%. Eventually, the retained capacity of 1.5SNF-high increased approximately 30% compared to 1.5LNF-low.

## 4. Conclusions

We investigated the effect of the cathode microstructure on the electrochemical properties of cells for a sodium metal chloride battery. The microstructure was designed to improve the cycle retention of a high-capacity Na/(Ni,Fe)Cl_2_ battery. The microstructure was designed by controlling the NaCl size and the granule density. In high-density granules, strong connections between metal particles were created owing to the high pressure, which was a key factor in the high cycle retention. However, high-density granules using large NaCl had a low porosity and a compressed Ni layer that obstructed the contact between the second electrolyte and cathode materials, which caused an insufficient initial capacity. On the other hand, using small NaCl mitigated the problems caused by the high pressure conditions, such as a compressed Ni layer on the NaCl surface, and the microstructure also had strong connections between Ni particles. Eventually, a Na/NiCl_2_ cell with high cycle retention was fabricated with small NaCl and high-density granules while minimizing the initial capacity loss. Finally, we applied the optimized conditions to fabricate cathode granules of a Na/(Ni,Fe)Cl_2_ cell, which preserved the advantages of this cathode, such as its high capacity and low cost. This cell successfully maintained a capacity of 224 mAh g^−1^ at the 100th cycle. These findings show that the cathode microstructure must be controlled for high cycle retention in a sodium metal chloride battery, and creating strong Ni connections is the most important point.

## Figures and Tables

**Table 1:** Variation in the charge capacity with different NaCl sizes and active metal ratios.

Cell Type	Cell Name	NaCl Size (μm)	Granule Density (%)	Ratio of Composition			Charge Capacity (mAh g^−1^)	
				Ni (wt.%)	Fe (wt.%)	NaCl (wt.%)	Cycle Number	
							2nd	80th
Na/NiCl_2_	2SN-low	<38	28	66.7	-	33.3	165	152
	2SN-high	(Small)	52				157	165
	2MN-low	38–150	32	66.7		33.3	174	169
	2MN-high	(Medium)	52				136	143
	2LN-low	150–300	37	66.7		33.3	184	166
	2LN-high	(Large)	55				120	140
Na/(Ni,Fe)Cl_2_	1.5LNF-low	150–300	-	30.1	30.1	39.8	263	172
	1.5SNF-high	<38	56				247	224

## Data Availability

The data presented in this study are available from the corresponding authors upon reasonable request.
